# Far-Transfer Effects of Strategy-Based Working Memory Training

**DOI:** 10.3389/fpsyg.2019.01285

**Published:** 2019-06-04

**Authors:** Sharon Chan, Ulrich Mueller, Michael E. J. Masson

**Affiliations:** ^1^University of Toronto, Applied Psychology and Human Development, Toronto, ON, Canada; ^2^Department of Psychology, University of Victoria, Victoria, BC, Canada

**Keywords:** working memory, strategy training, problem solving, executive function, far transfer

## Abstract

We assessed the transfer effects of training working memory strategies to a novel problem-solving task. Previous WM training studies have produced little evidence for transfer across contexts. In the current study, 64 6- to 9-year-olds were randomly assigned to one of four training conditions: semantic and rehearsal training, semantic training only, rehearsal training only, and treated control group. All training groups performed significantly better on the transfer task than the control group, but training groups did not differ significantly from each other. Implications of the findings for cognitive interventions and future WM training studies are discussed.

## Introduction

Working memory (WM) is a limited-capacity system responsible for temporary storage and simultaneous processing and manipulation of information (Baddeley, 2003; Müller and Kerns, 2015). WM has been linked to general intelligence and reasoning skills (Süß et al., 2002; Kane et al., 2004; Au et al., 2015), and shown to be predictive of academic outcomes such as school readiness and achievement (Bull and Lee, 2014; Müller and Kerns, 2015). Deficits in WM have also been implicated in neurodevelopmental disorders (Melby-Lervåg et al., 2012). Given the important role of WM capacity in cognitive processes and scholastic skills, considerable attention has been given to WM training with the goal of improving WM capacity (Karr et al., 2014; Melby-Lervåg et al., 2016; Weicker et al., 2016). A central issue in assessing the effectiveness of WM training concerns the transfer of training effects. If WM training produces improvements only in a narrow set of tasks that are highly similar to the trained task (near transfer), then it is hardly worthwhile investing resources in this type of training. Rather, to be considered effective, training effects should generalize to untrained tasks that are dissimilar from the trained task (far transfer; Barnett and Ceci, 2002). Furthermore, to be considered effective, training effects should also transfer temporally, that is, be maintained over time.

Independent reviews of training studies have arrived at different conclusions with regard to the effectiveness of WM training (see Klingberg, 2010; Shipstead et al., 2010; Morrison and Chein, 2011; Melby-Lervåg and Hulme, 2013; Karbach and Verhaeghen, 2014; Karr et al., 2014; Au et al., 2015; Melby-Lervåg et al., 2016; Weicker et al., 2016). Current research provides evidence for reliable short-term gains that generalize to somewhat similar WM tasks (intermediate transfer), yet there is no evidence that “working memory training convincingly produces effects that generalize to important real-world cognitive skills … even when assessments take place immediately after training” (Melby-Lervåg et al., 2016, p. 523; see also Shipstead et al., 2010; Melby-Lervåg and Hulme, 2013).

One reason for the inconsistent findings is the failure to pay attention to the distinction between untreated and treated (or active) control groups (Melby-Lervåg et al., 2016). In treated control groups, participants engage in activities that aim to provide equivalent exposure to non-experimental variables that may otherwise act as confounds, such as time spent interacting with the experimenter, or equivalent time looking at comparable stimuli. However, such filler activities lack the essential features characteristic of the training. In non-treated control groups, variables other than the intended training may be causing differences between groups. To illustrate the importance of treated control groups, consider a handful of studies measuring far-transfer training effects of non-verbal ability (e.g., Nutley et al., 2009; Jaeggi et al., 2011). In these studies, significant training effects were not detected when treated as opposed to untreated control groups were used. Furthermore, far transfer rarely has been documented in studies using treated control groups in combination with randomized designs (Wass et al., 2012; Melby-Lervåg et al., 2016). Thus, training studies should strive to include treated control groups to increase internal validity of the research design (Melby-Lervåg et al., 2016).

A further reason for inconsistent findings is that training methods may vary in their intended scope and specificity of training. WM training methods can generally be categorized as either *core-based* if they target domain-general abilities, or *strategy-based* if they target specific cognitive strategies that change how information is organized and encoded (Morrison and Chein, 2011). Core training methods are attractive because they are designed to target domain-general WM mechanisms. Such training would not be associated with a particular type of information or sensory modality, but would aid in the overall encoding, maintenance, and retrieval of information. Core-based training paradigms are necessarily complex because the training task must satisfy a long roster of criteria (see Morrison and Chein, 2011, for discussion), but such complexity often presents a challenge for task design and the interpretation of results when trying to identify specific mechanisms of change.

Another more problematic assumption made by advocates of core-based training is that because the training task is complex and involves core processes, observed improvements on the trained task equates to improvements in overall domain-general abilities. However, such effects can be interpreted as context-bound practice effects, and there is insufficient evidence that learning would transfer to new tasks that differ in presentation format.

In order to differentiate true training effects in core-processing ability from practice effects, one must be able to demonstrate transfer to a novel post-training task. The use of a novel post-training task, in turn, creates the problem that training and post-training tasks may not be tapping into the same underlying constructs. To circumvent this problem, researchers in cognitive training may adopt an alternative bottom-up approach by directly training specific strategies that are reflective of more efficient domain-general abilities, and which then can be applied to a variety of contexts. Strategies are effortful, goal-directed processes that enhance performance by facilitating information encoding, maintenance, and retrieval (St Clair et al., 2010; Morrison and Chein, 2011). In strategy-based training studies, participants are explicitly taught to use the strategy of interest and then encouraged to use and refine their mastery of specific skills in practice. In some situations, the specificity of strategy training is a grave limitation in itself, but in differentiating between strategies that promote a way of doing (e.g., remembering numbers in groups of threes to facilitate memorization), and those strategies that promote a way of thinking (e.g., chunking information at large makes memorization easier), strategies may serve as effective tools in new situations. Working memory strategies, in particular, can be used in different situations with analogous WM demands. For example, although rehearsal can be applied to remember discrete items in a list, it can also be used in combination with a mnemonic or acronym to remember more complex information such as a sequence of instructions or steps to a problem. Because of the omnipresence of WM demands in everyday situations, the training of WM strategies may benefit performance in multiple situations.

There are several additional advantages of strategy-based training over core-based training. First, core-based training programs tend to include a compilation of several tasks with the expectation that one training task, or some combination of training tasks, will produce an effect (e.g., Holmes et al., 2009). This results in a time-consuming and intensive endeavor that is not cost effective in time or resources (e.g., in the above study, training required 35 min per day for 20 days spread out between 5 and 7 weeks). Moreover, researchers are left to speculation at worst and theorizing at best, in pinpointing the specific components of the training program that are responsible for the training effects. By training specific strategies, it is easier to isolate and test the mechanisms or processes that account for improved performance.

Second, research shows that children with higher WM capacity differ from their peers in their patterns of strategy use. Although children with higher WM capacity may be benefiting from a combination of factors, individual differences have been shown to exist in children’s selection and implementation of strategies, and these individual differences in strategy use account for significant variance in performance on WM tasks (Engle et al., 1992; McNamara and Scott, 2001; Turley-Ames and Whitfield, 2003; Friedman and Miyake, 2004; Dunlosky and Kane, 2007; Kaakinen and Hyönä, 2007). The efficacy of strategy-based training may therefore lie in closing the gap between individuals with higher and lower WM capacity by bringing the strategy use of individuals with lower WM capacity on par with those with higher WM capacity.

Related to this idea are the two opposing hypotheses about the cause-and-effect relation of strategy use and WM capacity (Bailey et al., 2008). The strategy-as-effect hypothesis suggests that having higher WM capacity allows one to be more strategic in how information is processed and encoded, which in turn contributes to better performance on WM tasks. Alternatively, the strategy-as-cause hypothesis claims that strategy use is the direct cause of individuals demonstrating higher WM capacity. For example, a rehearsal strategy allows a person to retain more information, resulting in a higher span score. In support of the strategy-as-cause hypothesis, Dunning and Holmes (2014) found that WM training gains were mediated by spontaneous memory strategy use. Further support for this hypothesis comes from a study by St Clair et al. (2010) who found that comprehensive WM strategy training led to significant improvements in WM tasks assessing the phonological loop and central executive. Regardless of whether the strategy-as-effect or strategy-as-cause hypothesis is correct, remediation of strategy use could result in increases in WM capacity.

One pathway by which strategy use may facilitate performance on WM tasks involves decreasing the cognitive load placed on processing and encoding of information, thereby freeing up resources for storage. We argue that three developmental changes in strategy use, in particular, may impact efficiency in processing and encoding information.

First, several lines of research show that as children age, they increasingly organize information during both encoding and retrieval (Tulving, 1962; Shiffrin and Atkinson, 1969; Schleepen and Jonkman, 2012). Grouping and organization of information is particularly useful in facilitating delayed retrieval (Lange et al., 1990) and consolidation in long-term memory (Tulving, 1962; Schleepen and Jonkman, 2012). Interestingly, a recent study suggests that not all grouping strategies are equal: categorization based on semantic features (e.g., types of dogs) has been shown to improve memory performance more than categorization based on perceptual features or personal associations (e.g., animals that the child liked, disliked or feared) (Schelble et al., 2012). One possibility is that categorization provides more salient cues that link many concepts for quick retrieval. Children and adults with higher WM capacity were also found to use the classification strategy more often independently and spontaneously than peers with lower capacity (Rosen and Engle, 1997; McNamara and Scott, 2001; Schleepen and Jonkman, 2012). Some evidence points to more efficient patterns of strategy leading to better performance on tests of WM capacity, rather than higher WM capacity *per se*. For instance, Schelble et al. (2012) showed that children’s use of a semantic strategy made a stronger contribution in predicting retrieval performance than did their individual WM capacity scores. Moreover, although children with higher WM capacity have been found to be more strategic than children with lower WM capacity in free recall tasks, presenting participants with retrieval cues which prompted better strategy selection eliminated this difference (Unsworth et al., 2013). This finding suggests that effective organizational strategies such as semantic categorization could compensate for lower WM capacity in a demanding retrieval task.

A second developmental change in strategy use concerns the shift from non-verbal to verbal encoding by means of verbal or phonological rehearsal. Rehearsal is particularly useful when there is a delay between the presentation of information and recall, as rehearsal helps maintain and refresh information in verbal short-term memory (Morrison and Chein, 2011). There are two processes involved with rehearsal, the initial recoding of visual stimuli into a verbal format, followed by the rehearsal of recoded items in the phonological store; children may struggle with rehearsal by failing on the first or both of these steps (Flavell et al., 1966). Rehearsal develops gradually starting from about 5–6 years of age but more consistent use of this strategy is not apparent until about 6–8 years of age as noted by phonological similarity effects (lower recall of lists consisting of phonologically similar items) and articulatory suppression effects (reduced recall when required to repeatedly produce a task-unrelated verbalization while encoding target items) occurring only in older but not younger children (Henry et al., 2000; Lehmann and Hasselhorn, 2007; Henry et al., 2012).

Although the acquisition of rehearsal follows a developmental progression, rehearsal training has been shown to improve WM task performance in both developmentally delayed (Conners et al., 2008) and typically developing children and adults (Ford et al., 1984; Ornstein and Naus, 1985). Furthermore, the use of rehearsal as a memory strategy appears to be particularly beneficial for children with lower WM spans (Turley-Ames and Whitfield, 2003).

A third developmental shift in strategy use involves the transition from passive maintenance to active refreshing of information in WM. This shift occurs around 7 years of age (Camos and Barrouillet, 2011). According to the task-switching model (Towse and Hitch, 1995; Hitch et al., 2001), younger children fail to implement maintenance activities while performing a concurrent task, resulting in time-based decay of the memory trace. Instead, they passively hold items in memory without any attempt at active maintenance. Thus, their ability to hold items in memory is greatly affected by the duration of the delay period between presentation and recall. Older children have an increased capacity to control attention and monitor cognitive processes, allowing them to allocate attention during processing to reactivate, or refresh memory traces in real time (Camos and Barrouillet, 2011).

In the present study, we compare the independent and cumulative effects of two strategies (rehearsal and semantic organization) on WM capacity. The two strategies differ in terms of their mechanisms and advantages. The rehearsal strategy is useful for refreshing and maintaining unrelated information, but may be susceptible to distraction, and it is constrained by children’s short-term storage for auditory information. A semantic strategy in contrast, is less constrained by an individual’s short-term storage, and instead relies on the cued activation of associated networks stored in long-term memory. This strategy may be more effective in retrieving larger amounts of information that can be visualized, but requires more planning (e.g., the foresight and ability to categorize on multiple levels) and may also be susceptible to commission or intrusion errors.

### Current Study

The primary goal of this study was to examine whether strategy-based training would transfer to a novel problem solving task. The design of this study takes into account several important considerations including the use of treated controls, a strategy-based training paradigm, and the careful selection of strategies that map onto the identified developmental changes that children undergo as they move from being less efficient to more efficient strategy users. There were three training conditions and one control group. One group received rehearsal training (R), another group received semantic training (S), and a third group received training in both strategies (S+R). Children were trained in semantic organization and/or rehearsal with the expectation that both strategies would improve efficiency of processing, thus freeing up mental resources in WM for storage, with the result that WM capacity could be increased. By extension, increased WM capacity would produce improvements in problem-solving performance that incorporates a WM component.

As mentioned above, rehearsal and semantic categorization strategies have been independently used in previous training studies to increase WM capacity. However, these studies have typically involved older children, and did not assess their use in younger children who may not be spontaneously using these strategies very efficiently, if at all. Furthermore, no prior study has looked at the combined effect of rehearsal and semantic categorization training, nor have previous studies examined training of these strategies in the context of a far-transfer post-test.

We expected that children who received WM training would outperform children in the control group, as developmental research has shown that children between 6 and 8 years use these strategies with varying degrees of success. We further predicted that the combined training condition (S+R) would be more effective than the individual training conditions, given that children in the S+R condition would be provided with more tools to increase their WM capacity. Among the single-strategy conditions, we expected that semantic-strategy training would be more effective than verbal-rehearsal training because previous research has demonstrated that children and adults with higher WM capacity tend to use deeper encoding strategies that create meaningful networks between the items to be remembered (Friedman and Miyake, 2004; Dunlosky and Kane, 2007).

Far-transfer effects were assessed using a novel problem-solving task that was qualitatively different from the task on which children were trained. Performance on this problem-solving task was expected to improve with the use of trained strategies, as WM demands were embedded within the task, but children had to (a) realize on their own that strategies would be helpful to the task and (b) choose to use them under conditions of increased cognitive demand and interference. Careful attention was given to the use of appropriate control tasks to address previous concerns about the use of untreated control groups. Control tasks were selected to correspond to semantic and rehearsal training phases. These control tasks were comparable in time, type of stimuli involved, and level of mental stimulation to the training tasks. All groups therefore spent approximately the same amount of time interacting with the experimenter.

Additionally, we examined a near-transfer effect for the semantic categorization strategy (e.g., Black and Rollins, 1982). Specifically, we tested whether children who were trained in semantic categorization would use this strategy post-training in a free recall task that involved a new set of stimuli.

## Materials and Methods

### Participants

Sixty-five typically developing children aged 6–9 years were recruited from private and public schools within Victoria, BC, Canada. Flyers were distributed to children in school and interested children and parents contacted the researcher for participation. To ensure that participants could follow instructions, only children who had English language fluency and the absence of any developmental delay and/or learning disabilities as reported by their parents were included in the study. Written and informed consent was obtained from parents for children’s participation, and child assent was obtained verbally. Data from one child was excluded due to an inability to understand and follow the instructions. The 64 remaining children completed all the pretests, training phases, and post-test measures over a single 1.5-h session.

Sixteen participants were randomly assigned to each of four conditions, with the only requirement that the age distribution was kept relatively similar between groups. There were no significant differences between the mean ages across groups. The S+R group (*M* age = 7.5; males = 9) received training in both S and R strategies. Controls (*M* age = 7.4; males = 7) received only the filler tasks in place of both semantic and rehearsal training. The S group (*M* age = 7.2; males = 7) received semantic training and rehearsal control tasks. The R group (*M* age = 7.4; males = 9) received rehearsal training and semantic control tasks. Thus, each group of children received two sets of tasks that were comparable in administration time and complexity. While the S+R group received two training sets, the S and R group received one each of a training set and a filler set. Treated controls received two filler sets. Filler sets are described in detail below.

### Measures

#### Pretests

To assess whether any between-group differences existed on relevant WM and short term memory abilities which could potentially lead to differences in performance on the novel problem solving task, several pretest measures were administered including a visual memory task (memory for matrices), a verbal memory task (forward and backward digit span), as well as a free-recall task to examine use of clustering or organizational strategies prior to training. A full breakdown of the item-level questions and scoring criteria are provided in Appendix B.

##### Forward and backward digit-span tasks

In the forward digit-span task, children were asked to recall lists of digits. Numbers were read to children at a pace of one number per second, and were prompted to repeat the list in the same serial order with, “Ready? Go.” Children received a score of 1 for a correct response, and a score of 0 for an incorrect response. Points were summed for a total score. The task was discontinued after two consecutive scores of 0. In the backward-span task, children were asked to recall the list in the reverse order in which it had been presented, using the same prompt and scoring criteria.

##### Visual short-term memory (VSTM) task (Logie and Pearson, 1997; recall version)

The child was presented with a matrix pattern drawn on white cards in which half the squares, chosen randomly, were colored red. The pattern was displayed for 2 s and then removed, followed by a further 2-s delay during which the child was shown a blank white card. The child was then given an empty version of the same matrix and asked to point to the squares that previously were colored red. Matrices increased in size, with the proportion of red squares always fixed at 0.5. Children’s responses on each of the three trials of Matrices were recorded live by the experimenter on a blank grid. Correct squares were then tallied for each trial, and an average score was computed.

##### Free-recall pretest

The task was adapted from Black and Rollins (1982). Free-recall tasks are traditionally administered to adults in a written list format but given that some children in this age group would not be actively using verbal strategies and had limited reading skills, pictures were used instead. Colored photos were used instead of black and white line drawings to provide a more realistic and ecologically valid representation of objects (Moreno-Martínez and Montoro, 2012). Five cards each from four categories (insects, fruits, vehicles, and furniture) were chosen for use in the free recall task, for a total of twenty items. Different items were used in the post-test and the free recall post-test. High-frequency items previously used with children from each category were preferentially selected (Snodgrass and Vanderwart, 1980; Rabinowitz, 1984). Following the example of Black and Rollins (1982), duration of study time was determined by the child. At the end of the study period, the examiner collected the cards and asked the child to name as many items as possible. During recall, if the child appeared to run out of answers, they were prompted once with, “Can you remember any more?” before recall was terminated. The decision to allow the child to determine study time was also made with two considerations: firstly, that a timed study period would be stressful and anxiety-provoking for children and could impair their ability to remember, and secondly, that the vast majority of children in initial testing were able to self-report in a reasonable time frame as to when they were ready to have the cards taken away. The total number of correct responses was recorded live.

#### Training Tasks

The goal of this short-term intensive training was to increase children’s familiarity with and hone their correct use of strategies. Therefore, accuracy was not recorded or analyzed for the training tasks. Children were also encouraged to try as many times as possible until they arrived at the correct answer. Only one item was correct in any given array of objects. The duration of each section of training was kept as closely as possible to around 10–12 min, with control tasks timed for a similar duration.

##### Semantic-categorization training tasks

These tasks encouraged children to organize information based on their common abstract properties (e.g., things that hold liquid, things that fly, etc.). Children were first trained to think in terms of categories, and then to apply them strategically. Training involved two phases. In phase A, children had to make decisions about which object in a group did not belong with the others. For example, on one slide, children were shown a butterfly, beetle, spider, and banana. They were asked, “Which does not belong?” followed by, “What do the other ones have in common?” Nine training sequences were administered for Phase A of the training (see Appendix C for all training items). The number of items presented in each array ranged from 4 to 6 objects. Two levels of difficulty were administered. In the first five training sequences, objects in the same category shared the same identity (e.g., they are all insects, vehicles, furniture...). Children were then prompted that items in the final four sequences would be similar in ways that were harder to see (e.g., container/non-container, animate/inanimate, things that travel on land/water). Children were given the opportunity to discuss their ideas for each training sequence with the experimenter and were debriefed on all correct answers.

In phase B of semantic training, a scaffolded free-recall task was administered. Children were first prompted to sort the cards by their categories (fruit, insects, furniture, vehicles). Next, they were instructed to use the strategy of thinking about the similarity among items (“If you study the cards that are similar together, such as all the fruit together, it will be easier to remember them”). Black and Rollins (1982) had found this method of explanation to be most effective in encouraging children to adopt the category clustering strategy. The experimenter checked whether children could identify a few of the items that were similar and moved these cards closer together to better illustrate the grouping. Once it was clear that children had a grouping strategy in mind, they were given 3 min for recall. As accuracy was not measured during the training, children were given positive reinforcement for their attempts at using the strategy. Children were also debriefed on their performance and given feedback for correct application of the categorization strategy.

##### Semantic control tasks

In the first control task, children were given a regular deck of playing cards and asked to find all the cards that fit an arbitrary criterion of color and shape (e.g., all the red hearts, black clubs, etc.). In the second control task, the experimenter randomly selected a few cards from the free-recall deck (e.g., apple, ant, spider) and children were asked to tell a story about the items.

##### Phonological-rehearsal training

The goals of the rehearsal tasks were to train children in (1) recoding visual information into verbal information, and then (2) maintaining that verbal information in temporary storage through rehearsal. In the first phase of training children were asked to label a list of pictures out loud. They were then asked to rehearse the list until they felt ready to report the items without referring to the pictures. This recording of pictures into words followed by rehearsal was practiced over four trials of increasing list length, starting with three items and ranging up to seven items, with item length increasing by one item in each added trial (see Appendix C for full list). For example, on the first training sequence, children would see images of an ant, eye, and car. They were asked to label these objects and verbally rehearse them out loud, followed by several more repetitions either out loud or through inner speech. They were told to let the experimenter know when they were ready to have the pictures removed, and then repeated the items they had rehearsed. Effort was praised, as was successful memorization of increasingly longer sequences. In the second phase of rehearsal training, word lists were presented orally without the use of pictures, and children were tasked with recalling lists after a short delay. Children were then tasked with practicing this rehearsal strategy over five trials, starting with a sequence of three items and ranging up to a sequence of seven items. The sequence length increased by one item in each added trial. As the goal of training was mastery of the technique, children were allowed to repeat trials as necessary.

##### Rehearsal control tasks

Children were asked to read from a picture book with the help from the experimenter, or engage in a discussion about what they did on the weekend or about upcoming activities at home or school for the duration of approximately 10 min.

#### Post-tests

##### Problem-solving task

A problem-solving game was developed for this study. The most important function of the problem-solving post-test was to assess the cross-contextual far-transfer of any potential training effect. As such, novelty was a critical aspect of the task. Omitting to include a problem-solving *pretest* came with certain advantages and disadvantages, and the decision was ultimately made for several reasons: (a) Even though exposing children to a problem-solving pretest is often expected in a pre-post-test design, pretest administration has the disadvantage of introducing a practice effect, which reduces novelty of the task; (b) The average running time for this study was approximately 1.5 h. With the problem-solving task taking a large proportion of this time, adding a pretest would have necessitated a second testing session, which was not feasible at the time of data collection. Several safeguards were implemented to ensure as much as possible that groups had no significant differences at pretest: (1) A memory battery was administered at pretest (visual matrices, digit spans, free recall) to ensure that no between-group differences existed on a variety of potentially relevant memory abilities. These measures were then examined in relation to the problem-solving post-test in a subsequent regression analysis; (2) Near-transfer (recall post-test) and far-transfer (problem-solving task) are clearly distinguished in the results; (3) The use of random assignment usually safeguards against pre-existing group differences, and (4) The use of a treated control group with appropriate filler tasks ensured that pre–post differences would be related to intervention effects and not to unspecific factors (e.g., engagement with children). Overall, we believed that these measures compensated for the lack of the problem-solving pretest, while retaining the novelty effect for assessing far-transfer.

**FIGURE 1:**
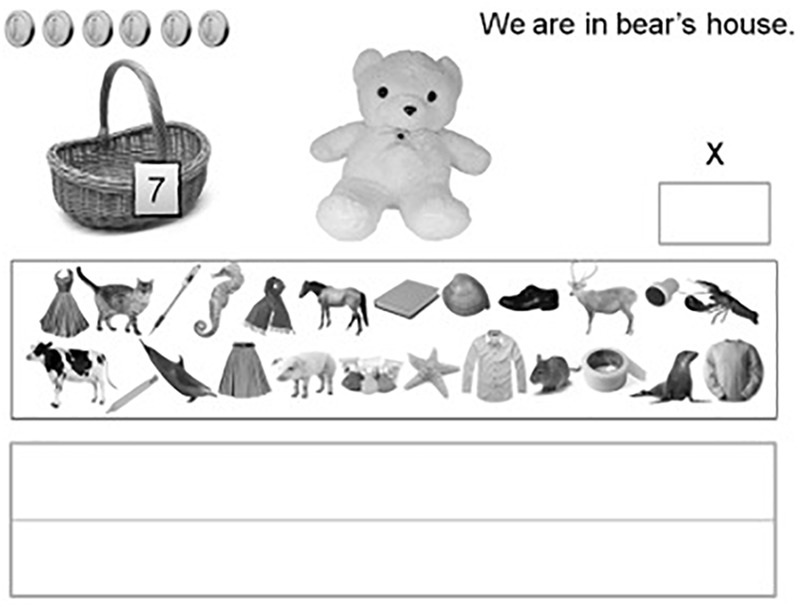
Initial set-up in bear’s house.

The problem-solving task was structured like a shopping game where the child had to retrieve items from a teddy bear’s list. It involved three adjacent rooms: two troll houses where children collected cards with pictures of items, and the bear’s house in between. The goal of the task was to retrieve all the cards on bear’s list while making as few errors as possible (see Appendix A for task instructions). Each child completed three different lists of items (three trials). Each trial consisted of 24 target items, with each item repeating only once across the three trials. There were a total of 48 possible cards, with 12 items of each category (Animate: aquatic animals, terrestrial animals; Inanimate: school supplies, wearable items). One troll housed animate categories and the other housed inanimate categories, but children were not told of this arrangement. Children were first introduced to the bear, and then to each of the trolls. Each troll had a coin bank for the child’s payment in order to open the box of cards. The experimenter enforced correct token use. To elicit strategy use, several constraints were put in place for the problem-solving task: (1) Children received only six tokens to pay the trolls. Each time a troll’s box was opened, a coin was forfeited. Once all the coins had been used, the trial was terminated, regardless of whether or not all cards had been collected. Thus, there were no explicit rules about when or how often the child could return to consult the bear’s list, nor any explicit penalty for selecting incorrect cards, but the limited number of tokens forced children to maximize the cards they would get with a visit to a troll. (2) Maximally seven cards were allowed to be kept in the basket at any given time. The number 7 was placed on the side of the basket, to serve as a visible reminder of this rule. This constraint ensured that children did not walk away with the entire deck of cards at once. During the collection process, children could collect cards in any order they liked. This allowed children to make plans about the best way to collect cards. (3) Upon returning to the bear’s house, the cards were placed on bear’s “shelf” (two empty marked-out rows) in the same order as bear’s list. This rule was designed to help children keep track of remaining cards. (4) Children were told that in order to win the game, all correct cards had to be retrieved while making as few mistakes as possible and using as few of the coins as possible. Children did not have access to the bear’s list while in the Trolls’ rooms and had to remember which items to retrieve. Figure 1 shows the setup of the list in bear’s house. Children were permitted to consult the list again when they returned to the bear’s house to place their collected cards. None of the children were given any explicit instruction that they should use a particular strategy or that the items could be sorted into categories.

We expected performance on this task to be improved by our specific training for several reasons. The 24-items presented in target lists would far exceed any individual’s maximum capacity, and children who received training in either or both strategies would be equipped with tools to improve WM at all stages including encoding, maintenance, and retrieval, an advantage that the control group would not have in such a demanding task. In addition, the task constraints, as well as children’s own WM capacity limits would be best overcome by the use of both strategies. For instance, a child could first plan to visit the room with the animate items, grouping animate items from the bear’s list using a rehearsal strategy, and then visit the room with the inanimate items on a separate trip, again using rehearsal for these items. Use of a rehearsal strategy alone meant that items from different categories rehearsed by the child would not be completed on the same trip, as each room housed only animate or only inanimate items. Similarly, children who used a semantic strategy only would not benefit from the use of rehearsal to maintain and refresh items in memory while shifting through the deck of cards and be prone to making more intrusion or commission errors.

For each of the three trials, the following outcomes were recorded: number of correct cards retrieved, number of errors, number of tokens used, and the number of cards retrieved for each token. Performance scores were computed as follows: total number of correct cards retrieved over three trials (maximum = 72), total number of errors over three trials, and total tokens used (maximum of 18 over three trials). A performance index was calculated by subtracting total errors (E) from total correct cards (C), divided by number of used tokens (T): (C-E)/T.

##### Post-training free recall

A post-training free-recall task was administered to all groups after the game as a near-transfer measure. The post-test used a different set of stimuli and categories (e.g., body parts, nature, instruments, kitchen utensils) than were used in the pre-test.

## Results

First, we compared the results of baseline WM and clustering pre-measures across groups. Second, far-transfer effects were compared across groups by looking at performance on the problem-solving task. Third, to evaluate near-transfer effects, groups that received semantic-categorization training were compared to those that did not in their performance on the post-training recall task. Finally, we evaluated the construct validity of the problem-solving task.

### Pretests

Prior to analyzing training effects, group performance on a variety of pretests was examined to ensure that there were no significant group differences in memory abilities which could have led to between group differences in our post-test. Group means for pretest measures can be found on Table 1.

**Table 1:** Group means for pretest measures.

*Groups*		*Matrices*		*Free recall (correct)*		*ARC*		*DS forward*		*DS backward*	
	*n*	* 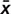 *	*S*	* 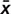 *	*s*	* 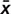 *	*s*	* 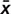 *	*s*	* 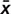 *	*s*
*S+R*	16	4.52	0.97	12.69	2.41	0.4	0.36	*5.69*	0.87	3.56	0.96
*Controls*	16	4.19	0.64	11.13	2.92	0.31	0.32	5.31	0.95	3.19	0.83
*S*	16	4.4	0.4	11.38	3.07	0.37	0.4	5.75	1.07	3.19	0.66
*R*	16	4.75	0.79	10.88	2.99	0.37	0.4	5.13	0.62	3.19	0.75


*ARC is a measure that reflects children’s use of the clustering strategy in the Free Recall task (see Senkova and Otani, 2012).*

Given that digit span scores are not expected to be normally distributed (Babikian et al., 2006), Kruskal–Wallis tests were used to test group differences on the digit forward and backward span total and longest span. The test revealed that the total scores for both forward, χ^2^(3) = 2.96, *p* = 0.40, and backward χ^2^(3) = 3.05 *p* = 0.38 digit span were not significantly different across groups.

Shapiro–Wilk normality tests suggested that matrices W(64) = 0.97, *p* = 0.119, number of cards W(64) = 0.97, *p* = 0.14 and ARC W(64) = 0.57, *p* = 0.57 at pretest of the free recall were normally distributed. Box’s M suggested that equal covariance matrices of the dependent variables can be assumed across groups *F*(9,41255.3) = 0.84, *p* = 0.58, and Levene’s test showed that variance of matrices *F*(3,60) = 1.32, *p* = 0.28 and number of correctly recalled cards *F*(3,60) = 0.25, *p* = 0.86 were assumed equal across groups. A MANOVA was conducted to test the between-group differences for matrices and the pretest free recall. Results using Pillai’s trace showed no significant between-group differences for these measures *F*(1.38,120) = 1.38, *p* = 0.23.

These findings suggest that there were no significant baseline differences between groups on the pretests of visual and verbal short-term memory and WM, as well as in the tendency to use a clustering/organizational strategy. Based on these findings it is reasonable to conclude that no group had any short-term or WM advantage compared to other groups prior to training.

### Near-Transfer Training Effects

We expected to see far-transfer effects only if near-transfer effects were first established, as more proximal transfer would predict more distal transfer. To reduce testing time, we prioritized the measurement of near-transfer to one task, specifically the free-recall task, because it was quick to administer and was a well-established measure for at least one of our trained strategies. Participants were grouped together depending on whether they received semantic strategy training (S+R and S) or not (R and control). The mean number of correctly recalled items in groups that had not received semantic training was 11.00 (*SD* = 2.91) at pretest and 10.47 (*SD* = 3.35) at post-test. The means of the groups that had received semantic training were 12.03 (*SD* = 2.8) at pretest and 13.16 (*SD* = 4.78) at post-test. A 2x2 repeated measures ANOVA revealed a significant main effect for the factor of semantic training, *F*(1,62) = 5.55, *p* < 0.05, $\eta_{p}^{2}$ = 0.08; participants who received semantic training did better on the free-recall task than those who did not receive semantic training. There was also a significant interaction between the within-subjects variable of time (pre- vs. post- test) and training condition *F*(1,62) = 4.19, *p* < 00.05, $\eta_{p}^{2}$ = 0.06, indicating a larger positive change in performance for the semantic training condition.

### Relationship Between Problem-Solving Task and Working Memory

The novel problem-solving post-test was designed such that it made demands on WM processes. Specifically, children were required to remember items, correctly select these remembered items from a series of stimuli including both targets and distractors, inhibit retroactive interference from previous sets of remembered items, and simultaneously hold the rules of the game in mind. To check whether the problem-solving task indeed made WM demands, we examined the correlations between the verbal and visual pretest WM measures and the problem-solving task. Forward digit span, *r*(62) = 0.28, *p* < 0.05, backward digit span, *r*(62) = 0.34, *p* < 0.01, and matrices, *r*(62) = 0.31, *p* < 0.05, all were significantly correlated with the problem-solving task. Next, we entered digit span forward, digit span backward, and Matrices as predictors of problem-solving performance into a regression model. This analysis showed that matrices and backward digit span tasks explained 15.9% of the variance in problem-solving performance (*R*^2^ adjusted = 0.159), *F*(3,60) = 4.96, *p* < 0.01, and significantly predicted problem-solving performance (β = 0.27, *p* < 0.05, and β = 0.28, *p* < 0.05, respectively). The forward digit span was not a significant predictor of problem-solving performance (β = 0.09, *p* = 0.50).

### Far-Transfer Training Effects on the Problem-Solving Task

For the problem-solving task, the performance index (C-E)/T was computed using the total scores ([total correct – total errors]/total tokens used) across three trials of the task. A univariate ANOVA with training condition as the independent variable was conducted to examine whether problem-solving performance differed as a result of which training group children were assigned. Results revealed a significant main effect for condition, *F*(3,60) = 3.04, *p* < 0.05, $\eta_{p}^{2}$ = 0.13. *Post hoc* analyses using least significant difference (LSD) revealed statistically significant differences in the problem-solving performance index (C-E)/T between the control group and each of the three training groups. As can be seen in Table 3, on average, the control participants collected fewer cards than any of the training groups. Importantly, medium to large effect sizes were found for all three comparisons made between treatment groups and the control group (see Table 2). The largest effect size was found for the difference between the control group and the group that received both interventions (S+R). The three training groups (S+R, S, R) did not differ significantly from each other.

**Table 2:** Mean differences between groups on problem-solving outcomes over all trials.

*Condition*		*Difference in correct cards*	*Difference in performance index*	*p*	*Effect size (d)*
				
*(I)*	*(J)*	*(I - J)*	*(I - J)*		
Controls	S + R	–14.63	–0.98*	0.006	0.97
	S	–8.69	–0.73*	0.038	0.63
	R	–12.06	–0.75*	0.035	0.74
S + R	S	5.94	0.25	0.48	0.27
	R	2.56	0.24	0.5	0.3


*^∗^Significant at *p* < 0.05; large effect size *d >* 0.8.*

**Table 3:** Group means for problem-solving outcomes.

*Groups*	*n*	*(C-E)/T*		*Correct cards Trial 1 (/24)*		*Correct cards Trial 2 (/24)*		*Correct cards Trial 3 (/24)*		*Total Correct (/72)*		*Total Errors*		*Tokens used*	
		* 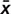 *	*s*	* 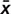 *	*s*	* 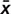 *	*s*	* 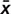 *	*s*	* 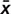 *	*s*	* 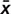 *	*s*	* 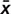 *	*s*
*S+R*	16	3.79	0.75	21.88	3.59	21.5	4.13	19.88	4.26	64.94	12.69	4.75	4.95	16.81	1.28
*Controls*	16	2.81	1.21	18.81	5.09	15.69	8.31	14.13	8.1	60.44	12.12	11.81	9.31	17.56	0.892
*S*	16	3.54	1.1	20.88	2.94	19.25	5.43	17.19	6.4	65.38	6.3	8.44	8.03	16.63	1.71
*R*	16	3.56	1.76	21.88	2.25	19.81	4.65	19	5.2	67.44	4.15	6.75	6.36	17.25	1.13
*Total*	64	3.42	1.02	20.86	3.7	19.06	6.1	17.55	6.42	64.55	9.66	7.94	7.63	17.06	1.31


*Total performance = (C-E)T.*

Due to the correlations between the pretest memory measures and the problem-solving post-test, a one-way ANCOVA was also conducted to examine whether post-test group differences remained when including the digit span tests, matrices, and free-recall pretest measures as covariates. Results showed a significant main effect for condition *F*(3,60) = 2.80, *p* < 0.05, $\eta_{p}^{2}$ = 0.13. *Post hoc* analyses again revealed that there were statistically significant differences in the problem-solving performance between the control group and the semantic training group (*p* < 0.05); the rehearsal training group (*p* < 0.05), and the S+R group (*p* = 0.05). No significant differences were found between the three training groups.

A breakdown of correct cards retrieved per trial is shown in Table 2, as well as means for total errors across three trials, and number of tokens used. No significant differences in token usage were found.

Perfect performance on this task would have involved collection of 72 cards in total, however no child collected all three lists in their entirety and without error, which demonstrated that the task was sufficiently challenging yet nuanced enough to show variability in performance across children.

## Discussion

The main finding of this study is that strategy-based training produced a far-transfer effect in a novel problem-solving task. The control group performed significantly worse on the problem-solving task than all three training groups. Most importantly, children were not coached into using specific methods to complete the problem-solving task, but had to recall and execute the strategies by themselves in a context that differed considerably from training (Barnett and Ceci, 2002). Surprisingly, there was no statistically significant difference in receiving combined training of both strategies compared to only one strategy.

A possible explanation for why there was no significant difference in problem-solving performance between the combined and separate training conditions is that the problem-solving task may not have been sensitive enough to capture the effects of the combined training. It is possible that these effects would have emerged if a more demanding outcome measure or a longer interval between training and test had been used. It is also possible that the training phase itself was too short. Cognitive training studies are generally time intensive, spanning multiple sessions over weeks. Training effects might have been amplified if there had been multiple training sessions to help consolidate learning.

Alternatively, children may not have benefited more from the combined training than from the single-strategy training because they may have used only one strategy although they had received training on both. Controlling the use of two strategies, in this case, first categorizing stimuli, and then rehearsing words recoded from pictures, would require the metacognitive ability to select and implement separate strategies in a logical sequence. This, in turn, requires the comparison and evaluation of different strategies, a mental task that may be difficult to do while attempting to implement a strategy (Whitebread, 1999; Touron et al., 2010). In a task that is cognitively demanding with multiple subordinate goals, children with limited cognitive resources will likely make less adaptive strategy choices (Imbo et al., 2007). Moreover, children with lower WM are more likely to experience a version of “utilization deficiency” (Bjorklund et al., 1994; Gaultney et al., 2005), such that, despite being instructed to use a particular strategy or seemingly comprehending the steps in its application, they fail at implementing the strategy due to limited cognitive resources. According to Lovett and Anderson (1996), available processing capacity in WM is a constraint that limits the amount of attention that can be distributed over concurrent tasks. Younger children who have lower WM capacity may not always be able to effectively implement strategies in spite of training. In conclusion, the effortful task of alternating and deciding between two strategies may actually impede the ability to use both, and if one strategy is easier to implement, a child may default to using only one. In addition to this possibility, the relatively small sample size in each condition would make it more difficult to detect differences between the training groups, especially if only a small effect size differentiates the groups.

An underpowered sample size was a notable limitation of this study. Due to the time intensive nature of pre- and post-test intervention studies, large sample sizes for these types of studies are achieved with some difficulty. The current study was also completed under a narrow time constraint, which made further data collection unfeasible. A one-way ANOVA would require a large sample size (*n* = 280) to detect a medium effect size (0.25) for four groups, using a 95% confidence interval. In the current study with a sample size of 65 the observed power for detecting a medium effect size was 0.338. Replication of this study with a sufficiently powered sample would ensure that a Type II error was not being made with respect to the lack of significant differences between the different training conditions.

The current findings also did not support our second hypothesis that children who were taught to use only a categorization strategy would outperform those who were taught only rehearsal. As previously mentioned, rehearsal may be less effortful than semantic categorization, and in cognitively demanding tasks, children tend to use less effortful strategies (Beilock and DeCaro, 2007). Our problem-solving task involved many rules in addition to memorizing items, and with these aspects demanding the child’s attention, the ability to use a more complex strategy efficiently may have been impaired. The near-transfer effect found in this study lends credence to this explanation. When comparing the group that received semantic training to the group that did not receive semantic training on the much less demanding free-recall post-test, those who received semantic training outperformed those who received rehearsal training.

Finally, the semantic training group may not have performed better than the rehearsal group because of intrinsic limitations to the categorization strategy. Semantic categorization is a good strategy for recalling a large number of items because of primed associations. However, it may actually impede performance in a task that places emphasis on accuracy for the very same reason. During free-recall tasks, participants occasionally make intrusion and repetition errors. In the current problem-solving task, retroactive inference between trials heavily impeded accuracy because targets remembered from a previous list may not have been targets on a current list. Accuracy was also an important component of the problem-solving task; if clustering both increased recall but also elevated the propensity for intrusion and repetition errors, the overall performance would be compromised. Future studies should seek to determine whether semantic strategies are more helpful when retroactive interference is reduced between trials. Retroactive interference could be removed by using each target for only one trial. A follow up study would also be improved by replication with more clearly distinguished categories, as the groups of aquatic and terrestrial animals may have been too similar for some children to fully benefit from using a categorization strategy. Nevertheless, despite the possible interference that children may have experienced while using a semantic strategy, the data showed that the semantically trained group still significantly outperformed the control group, suggesting that this strategy was overall successful in improving performance in a far-transfer task.

Lastly, it is important to distinguish between cross-temporal and cross-contextual transfer. Cross-contextual transfer is highly relevant to our understanding of how training effects can be transferred to real world environments. Although both cross-temporal and cross-contextual considerations are important, the focus of this study was on cross-contextual transfer, largely in part because of the narrow time constraints of data collection. Producing a sustained change is ideal, but longevity of a training effect may sometimes be secondary to the nature of change itself. Training which produces improvements in only a narrowly constrained set of outcomes may not be viewed as successful as training which produces improvement across multiple outcomes, particularly if those outcomes involve higher order cognitive process or more complex domains.

A challenge for the training literature has been to find a compromise in the similarities and differences between the trained and untrained tasks. On one hand, similarities between the tasks are part of the point of training studies: common elements must be identified and isolated in training, and improvement in these common elements are what we measure in transfer effects. If these common elements are unknown or inconsistent, predicting and implying transfer is impossible (Noack et al., 2009). On the other hand, if there is too much overlap between the trained task and post-test limits, then gains will likely be limited to near-transfer. In this study, we can clearly identify the common elements between training and post-test, as well as the overlapping elements between pretest and post-test. These common elements are in sum: a measurably capacity for short term storage of verbal and visual stimuli, a process whereby visual information is recoded into verbal information, the ongoing maintenance and refreshing of this information, and ways of organizing information effectively for more effective encoding and retrieval. Our training tasks focus on improving these elements, and the post-test recruits exactly these abilities.

At the same time, we acknowledge that it is debatable whether far-transfer has occurred in our study. The determination of whether far-transfer has taken place requires the administration of a series of post-test measures dissimilar enough from the trained tasks to suggest change at the level of broad abilities (Noack et al., 2009). We are less confident that our short training has made changes in any one broad domain. Nevertheless, the study demonstrated that the trained strategies could be used in a context where there was substantially higher cognitive load, as evidenced by the many other rules, constraints, and distractors, and where application of the strategies was less transparent and arguably more difficult to implement. Arguably, because of these features, the post-tests were at least somewhat dissimilar from the trained tasks, while retaining the common elements which underlie transfer.

### Near-Transfer Training Effects

A secondary goal of this study was to replicate near-transfer effects of semantic strategy training. We expected near-transfer effects for WM training more generally, because previous research has shown considerable evidence for near-transfer effects in training of executive functions (e.g., Klingberg et al., 2002; Turley-Ames and Whitfield, 2003; Li et al., 2008; Karbach and Kray, 2009). Results showed a significant interaction between type of training and pre- versus post-test free recall. Children who received semantic training showed a small but consistent improvement from pretest to post-test on the free recall task, whereas the performance of children who did not receive semantic training did not show this gain.

### Summary

The results of this study show that strategy-based WM training produced a far-transfer effect on a problem-solving task. Groups in all three training conditions outperformed control participants in problem solving. This research is important because it shows that children are able to generalize specific strategies to a completely novel problem-solving task with which they have no prior experience, and where no explicit instruction is given on how to complete the task. Moreover, not only were the test stimuli different from the ones used in training, the task demands that children were tested on (i.e., use of semantic categorization or rehearsal) were embedded in a game where other complex rules and hierarchical goals had to be kept in mind while remembering and implementing strategies. The positive findings of our relatively short training study stand in stark contrast to other research showing no evidence of far-transfer even in testing that immediately follows training. In our view, several factors contributed to the success of this training. First, we selected strategies that are developmentally appropriate in that these strategies were emerging but not yet mastered or used consistently by the target age group. Secondly, prior research has demonstrated these strategies to be used very effectively by individuals with higher WM capacity. In other words, children who do well in WM-related tasks employ exactly the strategies that were trained in the current study. Thirdly, in training these strategies we used very concrete, explicit instruction, and the ease of their use allowed them to be applied readily. Our finding shows that application is not limited to near transfer, but also generalizes to a new context.

Undoubtedly, more work is necessary to better understand the causal relation between components of WM and different types of higher-order abilities. Nevertheless, in examining our between-group differences, we found a large effect size for the difference between the combined training (Semantic+Rehearsal) condition and the control group, a marginally large effect size for the difference between Rehearsal training condition and the control group, and a medium effect size for the difference between the Semantic training condition and the control group. These medium to large effect sizes are exciting as they suggest that training simple strategies that focus on improving cognitive efficiency can potentially moderate children’s performance on higher-order tasks, particularly when task demands reflect components of training. The fact that combined training produced the largest effect size suggests that such training has the potential to have an additive effect.

Our training focused on practicing and fine-tuning strategies that emerge in children who are between 6 and 9 years old, suggesting that one direction of future research on WM training should focus on enhancing and scaffolding the effective use of cognitive strategies that are developmentally appropriate and contextually relevant. This study also replicated near-transfer effects, showing that those who received semantic training did better on a free-recall task after training whereas those who did not receive such training did not show improvement. This second finding is also important because it suggests that rehearsal and semantic categorization are two conceptually different mechanisms by which children can more effectively encode and maintain information in WM. Follow-up studies should further tease apart the unique contributions of these different strategies using more intensive training paradigms and larger samples. Future studies should be conducted to determine whether long-term gains can be produced by this type of WM training. In addition, there is a need to explore longer-term, distributed training paradigms, that take place over time and adjust for children’s changing competencies, as well taking into account individual differences in other factors related to task performance such as attention and motivation.

Finally, it is important to discuss several limitations of the present study. Despite the promising training effects, given the time constraint of data collection it was not feasible to conduct extended training over multiple sessions. Working memory training studies have typically involved several training sessions (about half an hour long each) over the span of a few weeks. Although the relationship between the quantity and effectiveness of training may be variable, it is likely that a more intensive training program would achieve longer-term training effects than a single session lasting half an hour as in the present study. In particular, children who were trained in two strategies may have benefited from more training sessions because of the complexity of mastering and applying two strategies.

Lastly, children’s baseline WM could be more precisely established in future studies to more clearly look at how children with low WM respond to training in comparison to children with high WM. It is also important that individual differences are not underestimated in any learning situation. Aside from baseline WM, other relevant factors such attention, emotional regulation, anxiety, or curiosity may have influenced children’s responsiveness to training and their ability to transfer training to a novel problem solving context. We also acknowledge that claiming far-transfer may be ambitious as it remains unclear to what extent our training could lead to permanent changes in a broad cognitive domain. Nevertheless, the study did demonstrate that trained strategies were used in the pursuit of a complex goal in a cognitively demanding context, in which children were not instructed to use these strategies, and in which, consequently, it arguably was more difficult to implement them.

## Data Availability

The datasets generated for this study are available on request to the corresponding author.

## Ethics Statement

This study was approved by the University of Victoria Human Research Ethics Board (Protocol No. 13-156) on May 13, 2013, and deemed minimal-risk.

## Author Contributions

SC contributed to all aspects of this study (conceptualization, design and methodology, data collection, interpretation, and write up). This study was designed and conducted as a part of the author’s M.Sc. requirements. UM contributed to all aspects of this study (conceptualization, methodology, interpretation, and write up) as the first author’s academic supervisor during the study’s completion. MM contributed to the methodology, interpretation, and write up as an integral member of the first author’s thesis committee.

## Conflict of Interest Statement

The authors declare that the research was conducted in the absence of any commercial or financial relationships that could be construed as a potential conflict of interest.
