# Supplementary material for: Far-Transfer Effects of Strategy-Based Working Memory Training
Source: Front Psychol. 2019 Jun 4;10:1285. doi: 10.3389/fpsyg.2019.01285 (PMC6558153; doi:10.3389/fpsyg.2019.01285)

**Appendix A: Task instructions**

*Matrices instructions*

Instructions:

*“*I’m going to show you some pictures of squares. Some squares are coloured red and some are white. Try your best to remember where all the red squares are. You need to look closely because you’ll only see the picture for a short time. After, you’re going to wait for me to give you the blank boxes and then you’ll point to where you think all the red squares were.”

*Free recall instructions*

Instructions:

Cards are displayed in four rows of five cards each, such that no two items form the same category are horizontally or vertically contiguous (see below before example). As each card is presented, the child names the item. In cases where the child does not name the item, the examiner will name it, and the child will repeat the name. During the study period, the child is shown that the cards can be moved, but no specific instructions are given.

“I am going to show you some pictures and you name them out loud as I put them down.” **(if child does not know an item’s label, experimenter label and ask child to repeat. Ask again after all items have been presented.)**

**(after presenting all the cards)** “Good, now I would like you to study them and try to remember as many of the items as you can. You can remember them in any order you like and you can also move these cards around however you like (demonstrate) if it helps you study. Take as long as you need to study and let me know when you are ready. When you are ready I will take the cards away and you will tell me all the ones you remember, okay?”

(remove cards, give child 3 minutes to answer. Encourage additional attempts with prompt, “Can you remember anymore?”)

*Rehearsal training phase A instructions*

Instructions:

“So, [name of child], you might have noticed that I’ve been getting you to remember a lot of things. One way of helping people to remember things is to repeat them over and over again in a list either out loud or inside their head. I’ll show you what I mean here (refer to the pictures on Powerpoint). So for example, if I were to say “car-hand-bee” over and over again, if I took away the pictures (change to blank screen), you’d probably still remember them, is that right? What were the items? Good. This will also help you remember the items if I ask you for them much later. I’d like you to use this way of remembering – by repeating them over and over again either out loud or inside your head, whichever you like. When you think you are ready, say, “ok!” and I will take the pictures away and ask you to tell me all the ones you remember.”

*Rehearsal training phase B instructions*

Instructions:

“Good job [name of child]! So now that you’ve had some practice and you’re very good at using this way of remembering, I’m going to make it a bit harder. This time I am not going to show you any pictures. I’m going to read you a list of items. Listen carefully and wait for me to finish the list. I’m going to read it twice to you. After, you’re going to repeat those items out loud or inside your head, and keep doing that until you see this “Go” appear on the screen and then you’ll tell me all the ones you remember, okay? (a Go is triggered on the screen after a 10-15 second delay.

*Problem solving task instructions*

Instructions:


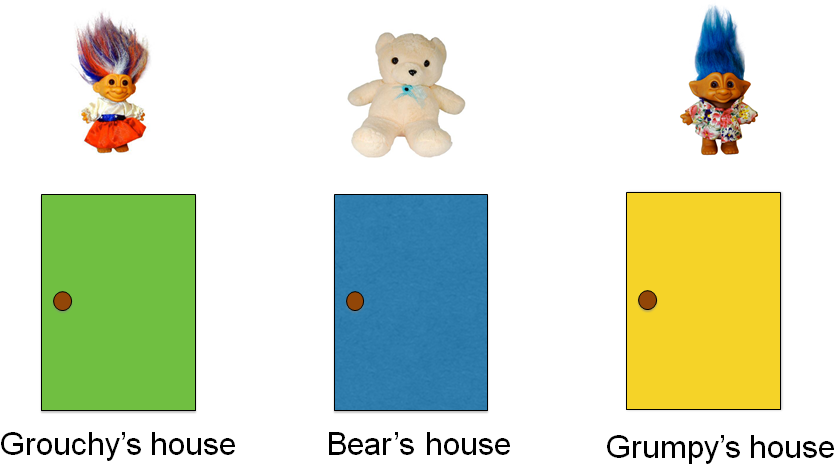


**(at bear’s house)** “Here is my friend Mr. Bear. Bear has a card for each of these pictures (point to items on list), but his cards have been stolen by two mean trolls named Grumpy and Grouchy. So [child’s name], it’s going to be your job to try and get back **all the cards on this list** for Bear. Can you do that? Let me show you where the trolls live.”


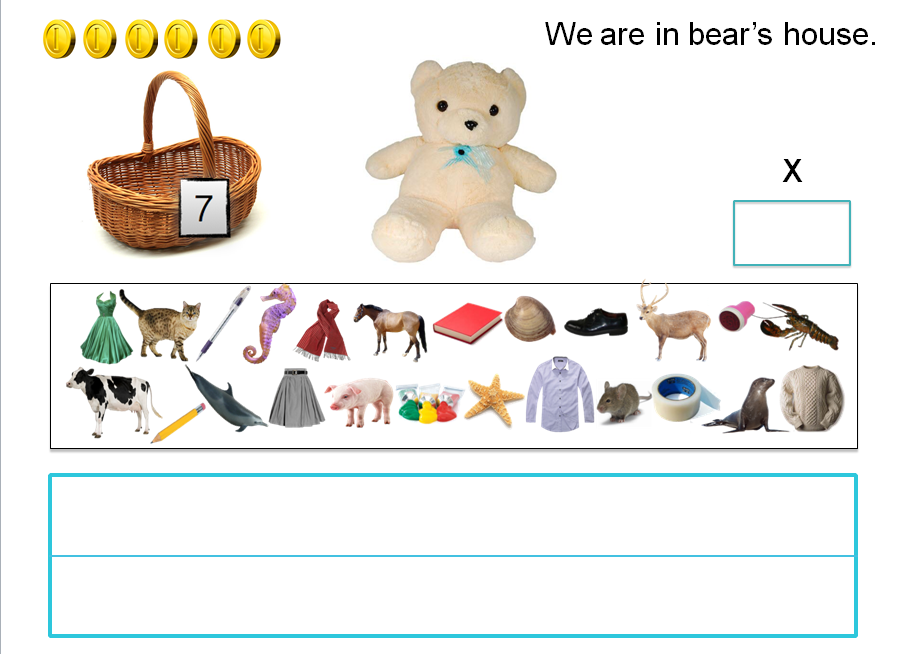


**(in Grumpy’s room)** “This is Grumpy and he has stolen some of Bear’s cards and mixed them up with all of his own cards and put them inside his box. Now before you can open this box you need to pay the troll one coin. (point to the coin bank) Always pay the coin first. This is going to be true for any of the trolls you visit in any of the rooms, you always need to pay them one coin before you open the box, okay? After you pay the coin, then you can open the box (demonstrate). Look through the cards carefully, pick the ones you need, and put the rest of the cards back into the box, and close the box.”

The same instructions and demonstration would be given to the child for the other troll in the green room before returning back to Bear’s house.

**(back at bear’s house)** “There are a few more rules I have to tell you about this game. You see this basket here? What does that say? (7) Good, this means that you are only allowed to have 7 cards in your basket at any time. You can have less, you can have 5, 6, and you can even have 7, but you can’t have more. So [child’s name] when you are collecting those cards from the trolls, make sure you count how many you plan to take away because you can only have up to 7. If there are cards you still want, you can put the ones in your basket down first and get the new cards on another trip.

I also have to tell you that you only have 6 coins. You have a lot of cards to get back but only these coins! So what this means is that you need to **think and plan carefully** about the best way to get these cards back.

After you bring the cards back to bear’s room, you’re going to put them on his two shelves (point to 2 marked out rows). They need to go in the same order as the list (point to items on list in order) but here’s the important thing: when you are collecting the cards from the two rooms, you can collect them in **any order you like.** (point to items on list in random) Whichever way you think is best. It’s only when they go back on the shelves that they have to follow the list.

If you accidentally get a card that is not on bear’s list, that’s going to be a mistake and that will go here (point to x box).

Now to win this game, you’ll have to try and get back all the right cards on bear’s list if you can, and try not to make mistakes. And you’re going to do this while trying to use as few of the coins as you can, alright? You can use all the coins if you need to, but try to use as few of them as you can.”

At this point the child is asked a few questions to confirm they understand the rules of the game:

“How many cards can you have in your basket at one time? (7)

Where do you put the pictures when you bring them back to bear’s house? (on the line)

Where do the mistake cards go? (x box)

What do you have to give the trolls every time **before** you can open the box? (give a token)”

“I will be waiting for you here to see which cards you bring back. Before you start you might want to have a good look at the list, and then you can start whenever you are ready.”

Appendix B: Pre-test scoring forms and stimuli

*Digit span forward*

| **Item** | **Trial Response** | **Score** |
| --- | --- | --- |
| 1 | 2-9 | 0 1 |
|  | 4-6 | 0 1 |
| 2 | 3-8-6 | 0 1 |
|  | 6-1-2 | 0 1 |
| 3 | 3-4-1-7 | 0 1 |
|  | 6-1-5-8 | 0 1 |
| 4 | 8-4-2-3-9 | 0 1 |
|  | 5-2-1-8-6 | 0 1 |
| 5 | 3-8-9-1-7-3 | 0 1 |
|  | 7-9-6-4-8-3 | 0 1 |
| 6 | 5-1-7-4-2-3-8 | 0 1 |
|  | 9-8-5-2-1-6-3 | 0 1 |
| 7 | 1-8-4-5-9-7-6-3 | 0 1 |
|  | 2-9-7-6-3-1-5-4 | 0 1 |
| 8 | 5-3-8-7-1-2-4-6-9 | 0 1 |
|  | 4-2-6-9-1-7-8-3-5 | 0 1 |

Total = LDSF =

*Digit span backward*

| **Item** | **Trial Response** | **Score** |
| --- | --- | --- |
| S | 8-2 |  |
|  | 5-6 |  |
| 1 | 2-1 | 0 1 |
|  | 1-3 | 0 1 |
| 2 | 3-5 | 0 1 |
|  | 6-4 | 0 1 |
| 3 | 5-7-4 | 0 1 |
|  | 2-5-9 | 0 1 |
| 4 | 7-2-9-6 | 0 1 |
|  | 8-4-9-3 | 0 1 |
| 5 | 4-1-3-5-7 | 0 1 |
|  | 9-7-8-5-2 | 0 1 |
| 6 | 1-6-5-2-9-8 | 0 1 |
|  | 3-6-7-1-9-4 | 0 1 |
| 7 | 8-5-9-2-3-4-6 | 0 1 |
|  | 4-5-7-9-2-8-1 | 0 1 |
| 8 | 6-9-1-7-3-2-5-8 | 0 1 |
|  | 3-1-7-9-5-4-8-2 | 0 1 |

Total = LDSB =

*Visual short term memory (Matrices) form.*

4-squares (2-span)


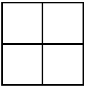

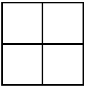

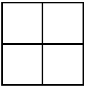


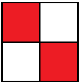

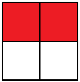

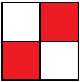


6-squares (3-span)


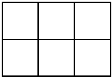

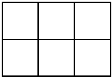

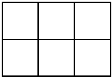


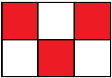

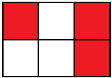

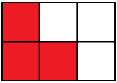


8-squares (4-span)


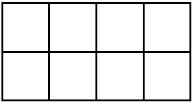

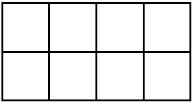

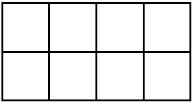


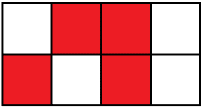

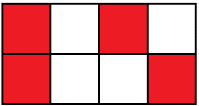

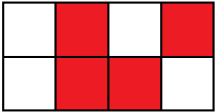


10-squares (5-span)


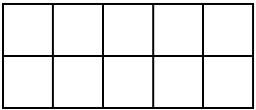

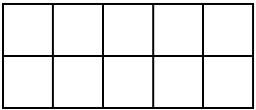

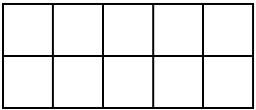


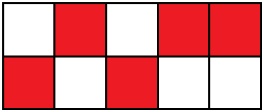

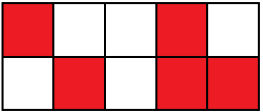

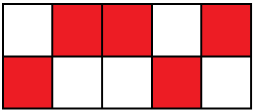


12-squares (6-span)


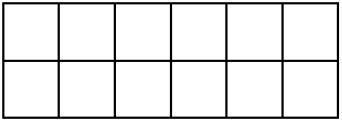

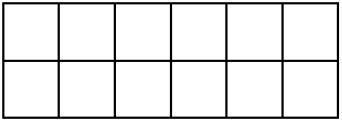

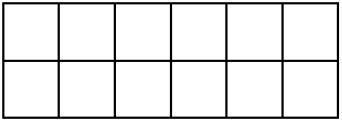


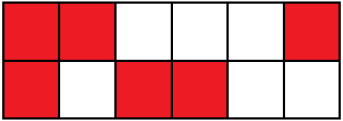

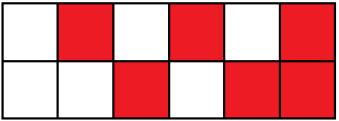

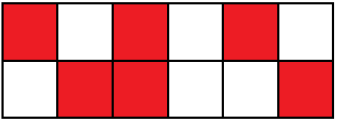


14-squares (7-span)


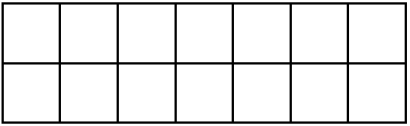

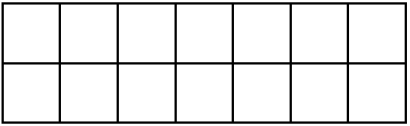

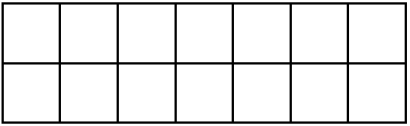


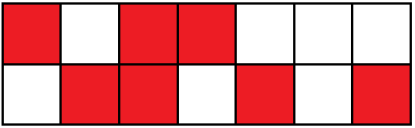

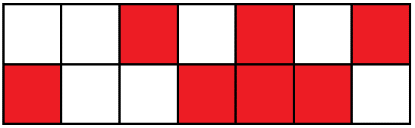

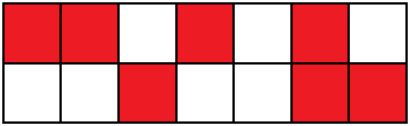


16-squares (8-span)


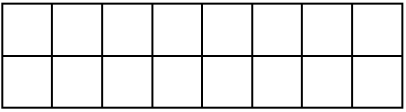

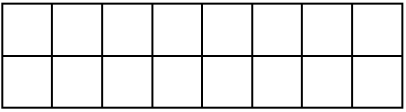

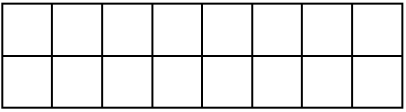


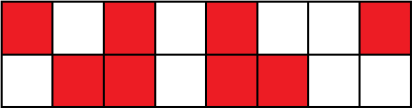

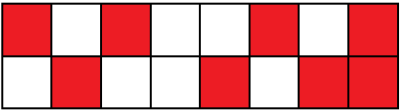

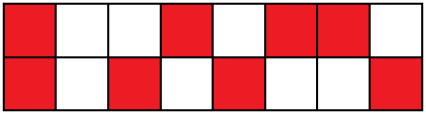


*Free recall Pre-test*

Correct = 1, Incorrect = 0
Categories: Fruit = 1, Insects = 2, Vehicles = 3, Furniture = 4

Participant Recall output (record in order of ouput):

|  | Item | Correct | Category |  | Item | Correct | Category |
| --- | --- | --- | --- | --- | --- | --- | --- |
| 1 | Banana | 1 | 1 | 11 | Car | 1 | 3 |
| 2 | Grapes | 1 | 1 | 12 | Plane | 1 | 3 |
| 3 | Orange | 1 | 1 | 13 | Bus | 1 | 3 |
| 4 | Cherry | 1 | 1 | 14 | Boat | 1 | 3 |
| 5 | Apple | 1 | 1 | 15 | Bike | 1 | 3 |
| 6 | Spider | 1 | 2 | 16 | Table | 1 | 4 |
| 7 | Ant | 1 | 2 | 17 | Chair | 1 | 4 |
| 8 | Bee | 1 | 2 | 18 | Lamp | 1 | 4 |
| 9 | Butterfly | 1 | 2 | 19 | Bed | 1 | 4 |
| 10 | Beetle | 1 | 2 | 20 | Sofa | 1 | 4 |

Appendix C: Training tasks stimuli

S1. CATEGORY INCLUSION DECISIONS

| Banana **(insects)** | Ant **(body parts)** | Fork **(vehicles)** |
| --- | --- | --- |
| Bike **(furniture)** | Leg **(kitchen utensils)** | Knife **(hold liquid)** |
| Ant **(fly)** | Clock **(have legs)** | Boat **(wheels/land)** |

Note: a group of images is displayed, correct answer is first word, category of other items is the second word in bold.

Level 1 Training Items:


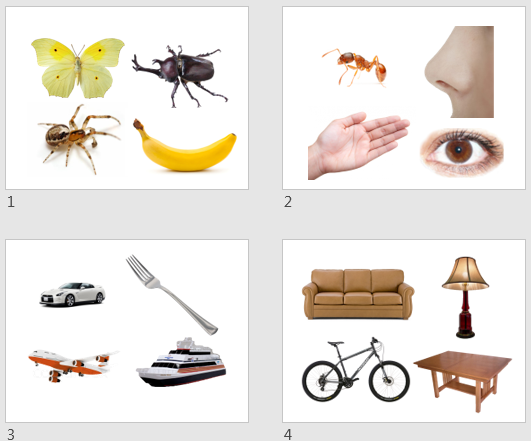


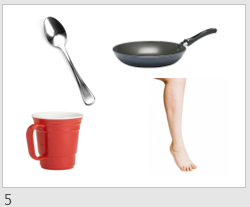


Level 2 Training Items


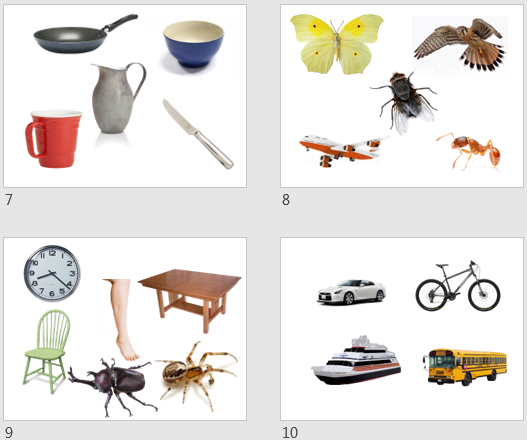


S2. TRAINED FREE RECALL (same as free recall pre-test)

REHEARSAL TRAINING LISTS

| Visual + Verbal |
| --- |
| Ant-eye-car  Lamp-bike-spoon-grape  Eye-boat-knife-bird-clock-drum  Fly-shoe-bear-hat-train-house-ball |
| Verbal presentation only |
| Chair-lamp-apple  Clock-cup-cherry-bus  Fork-cherry-spider-drum-bee  Knife-fly-train-clock-bed-lamp  Train-bed-house-car-beetle-spoon |

R1. READING from either Robert Munsch (younger kids) or A Series of Unfortunate Events (older kids)

EXAMPLE TRIANING ITEMS (Visual + Verbal training phase)

Example 1: 3-item list


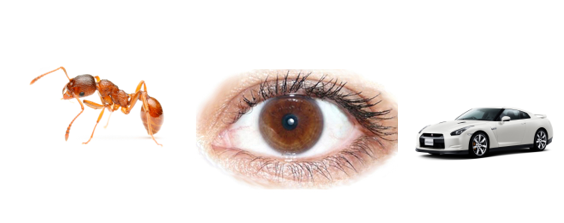


Example 2: 6-item list


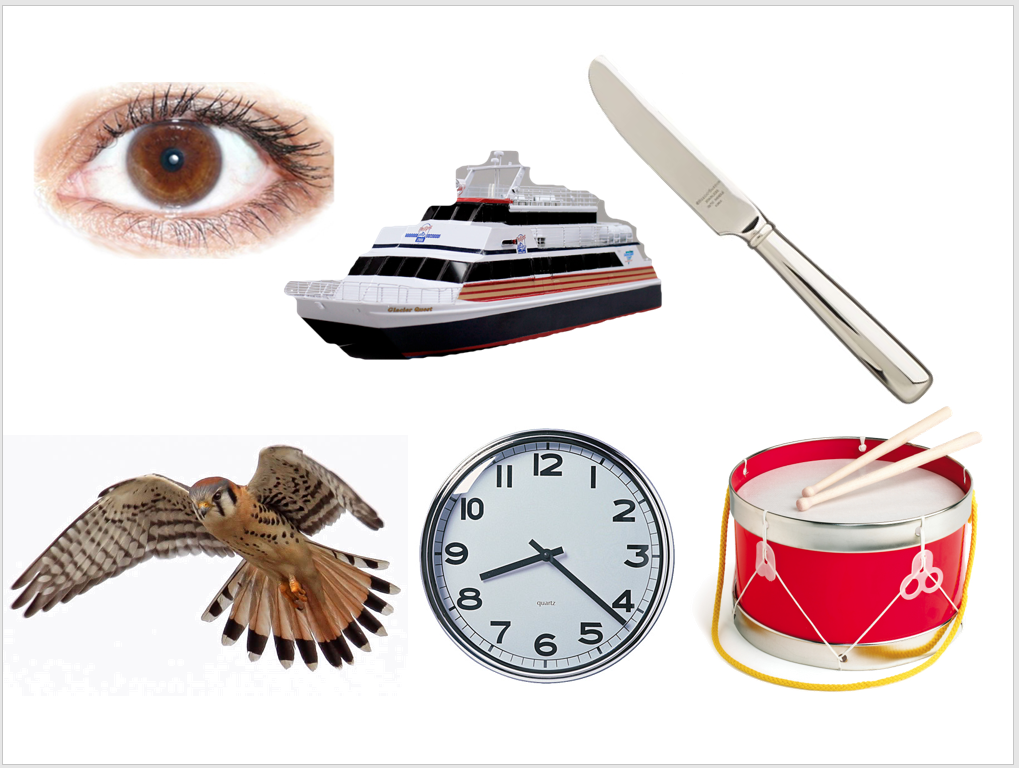

Supplement: Supplementary file 1 [file Data_Sheet_1.docx]
